# Mechanical Properties of Recycled Concrete with Carbide Slag Slurry Pre-Immersed and Carbonated Recycled Aggregate

**DOI:** 10.3390/ma18143281

**Published:** 2025-07-11

**Authors:** Xiangfei Wang, Guoliang Guo, Jinglei Liu, Chun Lv, Mingyan Bi

**Affiliations:** College of Architecture and Civil Engineering, Qiqihar University, Qiqihar 161006, China18053477239@163.com (J.L.); 02553@qqhru.edu.cn (C.L.);

**Keywords:** carbide slag slurry, carbonation, recycled aggregate, concrete, mechanical property

## Abstract

This research focuses on improving the characteristics of recycled concrete and utilizing solid waste resources through the combination of industrial waste pre-impregnation and the carbonation process. A novel pre-impregnation–carbonation aggregate method is proposed to increase the content of carbonatable components in the surface-bonded mortar of recycled coarse aggregate by pre-impregnating it with carbide slag slurry (CSS). This approach enhances the subsequent carbonation effect and thus the properties of recycled aggregates. The experimental results showed that the method significantly improved the water absorption, crushing value, and apparent density of the recycled aggregate. Additionally, it enhanced the compressive strength, split tensile strength, and flexural strength of the recycled concrete produced using the aggregate improved by this method. Microanalysis revealed that CO_2_ reacts with calcium hydroxide and hydrated calcium silicate (C-S-H) to produce calcite-type calcium carbonate and amorphous silica gel. These reaction products fill microcracks and pores on the aggregate and densify the aggregate–paste interfacial transition zone (ITZ), thereby improving the properties of recycled concrete. This study presents a practical approach for the high-value utilization of construction waste and the production of low-carbon building materials by enhancing the quality of recycled concrete. Additionally, carbon sequestration demonstrates broad promise for engineering applications.

## 1. Introduction

With urbanization accelerating and the building industry developing rapidly, the utilization of construction waste resources has emerged as a critical challenge for achieving sustainable development. Recycled aggregate concrete (RAC), a key product of construction waste recycling, can greatly reduce natural resource consumption by replacing natural aggregates [1,2]. However, the mechanical properties of recycled concrete produced with recycled aggregates are generally lower than those of natural aggregate concrete [3,4]. This is because the old mortar attached to the surface of recycled aggregates causes high porosity, high water absorption, and a weak interfacial transition zone, severely limiting its large-scale engineering applications. In recent years, many scholars have proposed methods to strengthen the properties of recycled aggregates, such as physical strengthening [5], chemical strengthening [6], and mineral phase modification [7]. However, these treatment approaches typically have disadvantages, such as high cost, high energy consumption, or low environmental friendliness [8,9,10,11,12].

Despite certain performance limitations, continuous innovations, such as improving RCA treatment and optimizing mix designs, can enhance recycled concrete to approach or even exceed traditional concrete performance, advancing this technology. Zhang [13] and Ollivier [14] believe that the flaws of recycled aggregates must be addressed to improve recycled concrete performance. To that end, numerous researchers have concentrated on improving the surface bonding mortar and the carbonation strengthening effect. To enhance recycled aggregates, researchers employed various pre-carbonation soaking methods. Tang [15] and Kong [16] used saturated Ca(OH)_2_ solution; Zhan [8], saturated lime water; and Xuan [17], an external calcium source with wet carbonation. All of these solutions improved the surface properties of the recycled aggregate by integrating more calcium sources, resulting in an effective approach for increasing the carbonation of the subsequent RCA.

To address high costs and environmental pollution from solution-modified recycled aggregates, this study proposes an innovative approach: we first pre-immerse recycled aggregates in carbide slag slurry and then carbonate them, leveraging the synergy of industrial solid waste utilization and CO_2_ capture. Carbide slag is a calcium-rich alkaline slag created during acetylene synthesis. Its principal component is Ca(OH)_2_, which has a high calcium content, tiny particle size, great dispersion, large specific surface area, and strong water solubility [18,19]. Pre-impregnation of recycled aggregates with carbide slag slurry can efficiently supply the calcium source necessary for the carbonation reaction, strengthen the carbonation effect, and improve aggregate performance. Carbonation mixes CO_2_ with calcium-based compounds to generate CaCO_3_, which densifies aggregate structures and efficiently fixes CO_2_ [20]. The synergistic effect of carbide slag slurry pre-impregnation and carbonation conditioning overcomes the limitations of traditional single modification technology, resulting in the synergistic strengthening technology of carbide slag slurry pre-impregnation–carbonation conditioning. This technology offers three key advantages. First, the carbonation product fills aggregate pores and enhances interfacial bonding, significantly improving the aggregate’s physical and mechanical properties. Second, it utilizes industrial waste slag and sequesters CO_2_, achieving both waste treatment and carbon emission reduction. Third, the process operates under mild conditions, delivering economic and environmental benefits.

However, due to its complicated chemical makeup, carbide slag may have an impact on the carbonation process and concrete properties. This study confirms the feasibility of carbide slag slurry pretreatment and carbonation for recycled aggregates through systematic experiments. It analyzes the carbonation reaction mechanism, clarifies how carbonation products affect aggregate performance, and provides theoretical support for the technology’s application. This research aims to analyze the mechanism of carbide slag slurry pre-impregnation–carbonation synergistic modification on the performance of recycled aggregates and to clarify how it improves the performance of recycled concrete. Compared to earlier studies, this report focuses on three key aspects: (1) proposing a novel method using carbide slag and CO_2_ to co-modify recycled aggregate, establishing quantitative relationships between impregnation–carbonation parameters and aggregate performance; (2) revealing how the calcium migration–carbonation coupling reaction reconstructs the aggregate–new mortar interface; (3) analyzing and verifying the synergistic enhancement effect of this technology on recycled concrete performance. This study establishes a technically synergistic system for solid waste resource utilization, CO_2_ sequestration, and cost control through in-depth analysis of reaction mechanisms and the construction of process flow models. This integrated approach achieves dual optimization of economic and environmental benefits, ultimately transforming low-value recycled aggregates into high-value-added green building materials.

## 2. Materials and Methods

### 2.1. Materials

#### 2.1.1. Cementitious Material

The cementitious material utilized in the test was P.C 42.5 composite silicate cement, and all of the indexes met the Chinese standard GB 175-2023 [21]. The chemical composition parameters of cement are provided by the manufacturer. See Table 1 for specific data.

#### 2.1.2. Aggregate

Xinyu Cement Products Co., Ltd. (Qiqihar, China), provided both natural coarse aggregate (NA) and recycled coarse aggregate (RCA). The natural coarse aggregate was granite, while the recycled coarse aggregate was obtained from demolished concrete wastes after crushing, cleaning, and screening. Natural coarse aggregate and recycled coarse aggregate were mixed 1:1 to produce coarse aggregate with particle sizes ranging from 5–10 mm to 10–20 mm. Aggregate property tests were conducted in triplicate on 3 kg samples for each aggregate type and size fraction, with the reported results representing the average values. All RCA utilized in this study originated from a single, homogenized batch. Table 2 shows the performance of the aggregate.

**Figure 1:**
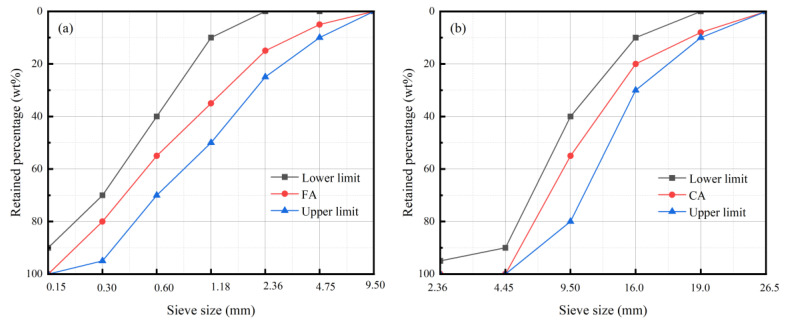
Grain size distributions of (**a**) fine aggregate and (**b**) coarse aggregate.

The experiment used local river sand with particle size ≤ 5 mm, fineness modulus 2.6–2.8, and particle size distributions of fine aggregate (FA) and coarse aggregate (CA), as shown in Figure 1.

#### 2.1.3. Carbide Slag

Heilongjiang Haohua Chemical Company (Qiqihar, China) donated the carbide slag used in this experiment, which is industrial waste created during the acetylene gas production process. Following the first crushing method, carbide slag was formed, and the carbide slag needed for the experiment was acquired after grinding to a fineness of 300 mesh. Table 3 shows the chemical composition of carbide slag.

#### 2.1.4. Water

The water utilized in this experiment is the same as that used by residents of Qiqihar City in Heilongjiang Province.

### 2.2. Experimental Program

#### 2.2.1. Preparation of Carbide Slag Slurry

In this experiment, five carbide slag slurries with mass fractions of 10%, 20%, 30%, 40%, and 50% were prepared strictly to the exact proportions. During the preparation process, continuous stirring was used to achieve homogeneous solid–liquid mixing, and the 30% mass fraction of carbide slag slurry was determined to be the saturated concentration via rheological property tests. This carbide slag slurry was used in all subsequent experiments to pre-immerse recycled aggregates to ensure the consistency and accuracy of the experimental conditions.

#### 2.2.2. Pre-Impregnation Treatment of Recycled Coarse Aggregate

The carbonation effect of recycled aggregate is closely related to its water content; hence, the water content of the aggregate during presoaking must be taken into account throughout the presoaking process. Ying Jingwei [22] investigated the carbonation effect of different aggregate water concentrations and found that RCA with moisture contents of 60–70% is more conducive to the carbonation reaction. As a result, in this experiment, RCA was first immersed in a 30% concentration of carbide slag slurry prepared in advance for 24 h. After draining, the aggregate was transferred to a thermo-hygrostat maintained at 20 °C and 65% relative humidity for 24 h static curing before use. To ensure that the water content of recycled aggregate before carbonation was consistent across all experimental groups, the control group was soaked in water, and the rest of the process was the same as for the treatment group.

#### 2.2.3. Carbonation of Recycled Coarse Aggregate

The carbonation effect is influenced by multiple factors, including temperature, humidity, CO_2_ concentration, and CO_2_ partial pressure. Ying [22] conducted a systematic study on the carbonation effect of recycled aggregates under different carbonation settings. After thoroughly considering carbonation efficiency, environmental friendliness, and energy efficiency balance, the optimal carbonation conditions were determined as follows: a carbonation temperature of 20 °C, a relative humidity of 65%, a CO_2_ concentration of 60%, and a CO_2_ partial pressure of 0.1 MPa. Therefore, the carbonation conditions established in Ying’s research were adopted for the carbonation test in this experiment.

**Figure 2:**
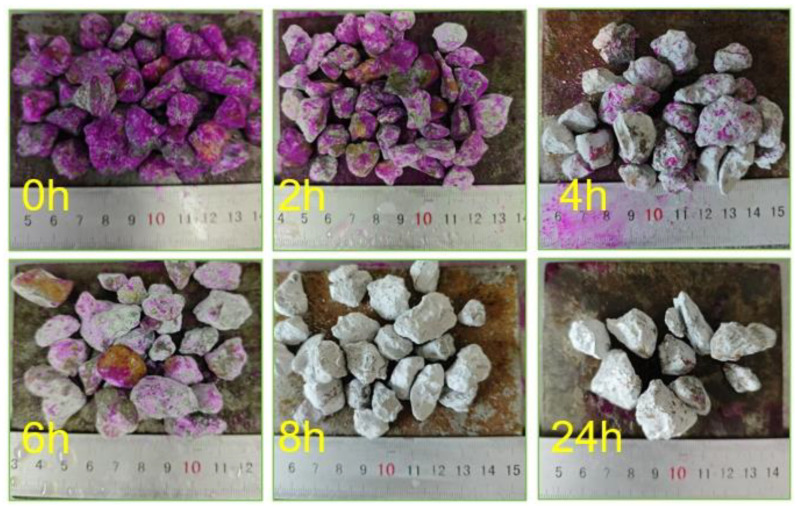
Reaction of CSS pre-immersed recycled aggregates with phenolphthalein for varied carbonation durations.

In addition to the carbonation conditions mentioned above, the carbonation duration also significantly influences the carbonation effect. As this experiment first proposes the use of CSS pre-immersed recycled aggregate, there is a lack of reference to previous research data to accurately determine the completion time of the recycled aggregate carbonation. Therefore, six groups of CSS pre-immersed recycled aggregate (500 g each) were prepared and subjected to carbonation treatment for 0, 2, 4, 6, 8, and 24 h under established conditions. Color change was used to indicate the degree of carbonation, where the aggregate was considered carbonated if no red color appeared after phenolphthalein indicator spraying. As shown in Figure 2, no red color appeared on the aggregate after 8 h of carbonation. By this time, the carbonation reaction had exhausted the OH^−^ on the aggregate surface, preventing it from reacting with the phenolphthalein reagent to form a red product. The experiment determined that the recycled aggregate had reached a state of complete carbonation after 8 h.

**Figure 3:**
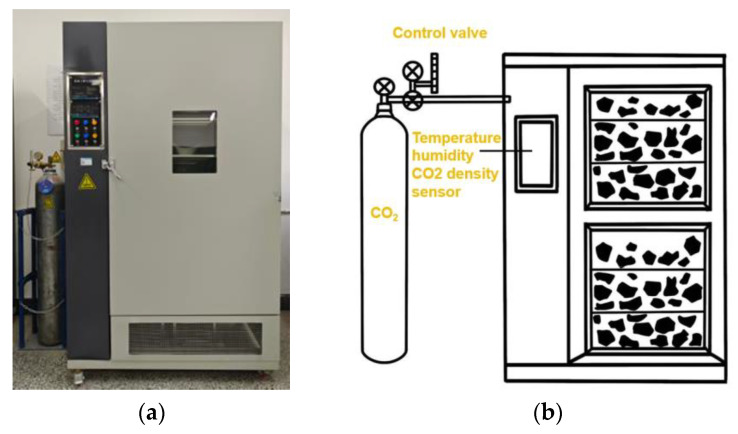
Carbonation device: (**a**) actual carbonation device; (**b**) schematic diagram of carbonation device.

In summary, the carbonation conditions of this experiment were determined as 65% relative humidity, 60% CO_2_ concentration, 20 °C carbonation temperature, 0.1 MPa CO_2_ partial pressure, and 8 h carbonation duration. The carbonation device is shown in Figure 3.

#### 2.2.4. Specimen Preparation

According to the Chinese standards (GB/T 50010-2010 [23]) and (GB/T 50080-2016 [24]), four groups of concrete specimens were prepared for this test, including natural aggregate concrete (NAC), 30% substitution rate recycled aggregate concrete (RAC), clear water pre-immersed carbonated aggregate recycled concrete (D-RAC), and CSS pre-immersed carbonated aggregate recycled concrete (C-RAC). The NAC group was designed with a target strength grade of C30, as defined by the Chinese Code GB 50010, which stipulates that the characteristic compressive strength at 28 days is ≥30 MPa. All groups were prepared with a water-to-cement ratio of 0.5 and sand ratio of 0.4. In this context, “FA” denotes fine aggregate. The specific test mixes are shown in Table 4.

### 2.3. Experimental Methods

#### 2.3.1. Aggregate Physical Properties

Following the Chinese standard GB/T 14685-2023 [25] Crushed Stone and Pebble for Construction, this experiment systematically determined the water absorption rate, apparent density, and crushing value indexes of recycled coarse aggregate (RCA) before and after carbonation treatment. During the experiment, three groups of samples were selected each time for independent testing, and data were obtained through standardized operations. The arithmetic mean of the three groups of experimental results was finally taken as the measured value of each index to ensure the reliability and representativeness of the data.

#### 2.3.2. Mechanical Properties

According to the Chinese standard GB/T 50081-2019 [26], the universal testing machine was used to carry out the mechanical property test of concrete samples. The 100 mm in side cubic samples were used for the compressive strength test and the splitting tensile strength test. The 100 mm × 100 mm × 400 mm prismatic samples were used for the flexural strength test. A total of 20 groups of 100 mm in side cubic samples (for compressive and splitting tensile tests) and 8 groups of 100 mm × 100 mm × 400 mm prismatic samples (for flexural strength tests) were prepared for the test, with three parallel samples set in each group, and the test results were the average values of the test values of the three samples in the same group.

#### 2.3.3. Microstructural Analysis

(1) XRD experiment

The specimens were dried in an oven at 65 °C for 24 h. Prior to XRD analysis, the samples were sieved to ensure a particle size of less than 80 μm and then scanned using a Rigaku Smart Lab diffractometer (Cu anode) (Tokyo, Japan) at a scan rate of 10°/min over a 2θ range of 5° to 60°.

(2) SEM scanning

Aggregate samples smaller than 10 mm were sanded, cut, and dried in an oven at 65 °C until they reached a constant weight, after which the micro-morphology of the samples was studied using SEM.

(3) EDS scanning analysis

Carbonated RCA was analyzed using EDS point scanning to compare the elemental content of various treatment methods and the outcomes of their elemental alterations.

## 3. Results and Discussion

### 3.1. Effect of Pre-Immersed CSS Carbonation on Physical Properties of Recycled Coarse Aggregates

#### 3.1.1. Water Absorption Rate

**Figure 4:**
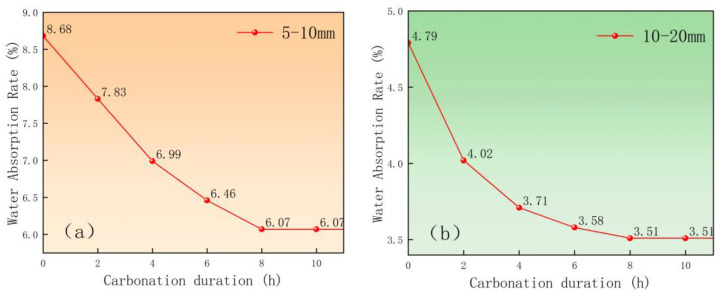
Effect of carbonation duration on water absorption of CCS pre-immersed recycled aggregates: (**a**) 5–10 mm C-RCA, (**b**) 10–20 mm C-RCA.

To comprehensively evaluate the effect of carbonation time on the water absorption rate of recycled aggregate, this experiment was carried out to research the water absorption rate of recycled aggregate at various carbonation times, and the findings are given in Figure 4. From the data in the figure, it can be seen that in the first two hours of carbonation, the water absorption rate of the recycled aggregate decreases the most, indicating that the carbonation reaction rate is the fastest in the first two hours. When the carbonation time is between 2 and 8 h, the water absorption rate continues to decrease with the prolongation of time, but the rate of decrease slows down compared with the initial stage, indicating a tendency to stabilize. This event shows that the carbonation reaction is essentially complete within 8 h, which is consistent with the findings of the phenolphthalein test in Section 2.2.3.

**Figure 5:**
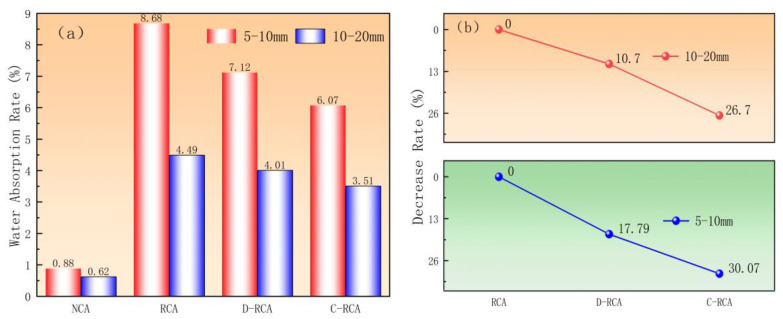
Effect of CSS pre-immersed carbonation treatment on water absorption of recycled aggregates: (**a**) water absorption rate, (**b**) decrease rate.

Meanwhile, to thoroughly investigate the effect of CSS pre-immersed carbonation treatment on the water absorption performance of recycled coarse aggregate, this study measured the water absorption of CSS pre-immersed carbonated recycled aggregate and compared it with that of natural aggregate, untreated recycled aggregate, and recycled aggregate treated with direct carbonation, as shown in Figure 5. As shown in Figure 5a, the water absorption rate of aggregates with a diameter of 5–10 mm was 8.68% when not carbonated, which decreased to 7.12% after direct carbonation and further decreased to 6.07% after CSS pre-impregnation. The initial water absorption rate of 10–20 mm diameter aggregates was 4.49%, which decreased to 4.01% and 3.51% after direct carbonation and CSS pre-impregnation, respectively. The CSS pre-impregnation–carbonation technique outperforms direct carbonation in reducing the water absorption of aggregates with various sizes. The mechanism can be attributed to the fact that calcium hydroxide in the CSS reacts with CO_2_ via carbonation, and the generated CaCO_3_ crystals and silica gel realize the dense reconstruction of the aggregate microstructure through the synergistic effect of physically filling internal pores and chemically bonding microcracks in the transition zone between the old mortar and the CSS. This is consistent with the findings of Zhan [27].

As shown in Figure 5b, for C-RCA, the water absorption reduction after carbonation was 30.07% and 26.7% for aggregates with sizes of 5–10 mm and 10–20 mm, respectively. For D-RCA, the corresponding reductions were 17.79% and 10.7%, respectively. These results indicate that carbonation had a more significant effect on reducing the water absorption of recycled aggregates, especially the particle size of 5–10 mm, which is consistent with the findings of Lu [28]. This phenomenon can be attributed to the higher adhesion rate of old mortar on smaller-sized aggregates, and their larger specific surface area results in a greater effective reaction area between Ca(OH)_2_ in the carbide slag slurry and CO_2_. This enhances the reaction kinetics of carbonation, thereby strengthening the improvement in water absorption performance [29].

#### 3.1.2. Crushing Value

**Figure 6:**
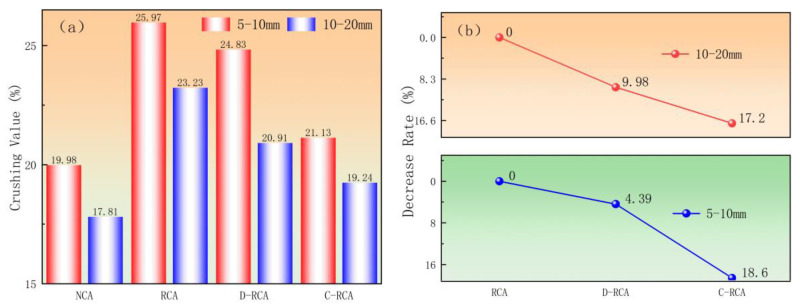
Effect of CSS slurry pre-immersed carbonation treatment on the crushing value of recycled aggregates: (**a**) crushing value, (**b**) decrease rate.

In this study, the crushing value of recycled aggregate before and after treatment was evaluated, and the test results of C-RCA and D-RCA were compared and assessed with RCA, as shown in Figure 6. As shown in Figure 6a, the crushing value for C-RCA 5–10 mm and 10–20 mm particle size aggregates fell from 25.97% and 23.20% to 21.13% and 19.24% before treatment, while for D-RCA 5–10 mm and 10–20 mm particle size aggregates it reduced from 25.97% and 23.20% to 24.83% and 20.91%. Figure 6b also shows that for C-RCA, the decrease in crushing value after carbonation is 18.6% and 17.2% for 5–10 mm and 10–20 mm size aggregates, respectively, and for D-RCA, the decrease in crushing value after carbonation is 4.39% and 9.98% for 5–10 mm and 10–20 mm size aggregates. It is clear that the carbonation of aggregates after CSS pre-immersed treatment improves their performance more significantly. The mechanism can be explained in the following two ways: Firstly, carbonation causes CaCO_3_ crystals to fill the previous mortar’s interfacial transition zone (ITZ), pores, and microcracks, resulting in a more dense microstructure. According to Vargas P [30], this increases the microhardness of the old mortar and the aggregate’s resistance to external crushing. Secondly, CaCO_3_ precipitation in RCA pores optimizes aggregate microstructure and reduces crushing value through physical filling and chemical cementation. Xuan [9] obtained similar results by investigating the carbonation of recycled materials.

#### 3.1.3. Apparent Density

**Figure 7:**
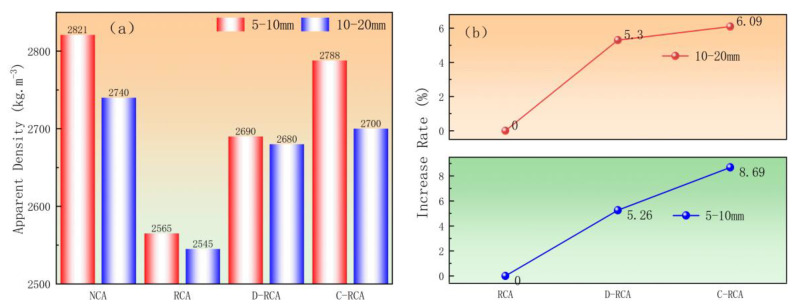
Effect of CSS pre-immersed carbonation treatment on the apparent density of recycled aggregates: (**a**) apparent density, (**b**) increase rate.

The effect of pre-impregnation–carbonation treatment on the apparent density of recycled coarse aggregate is shown in Figure 7. As indicated in Figure 7a, the apparent densities of 5–10 mm and 10–20 mm aggregates increased from 2565 kg/m^3^ and 2545 kg/m^3^ to 2788 kg/m^3^ and 2700 kg/m^3^, respectively; for D-RCA, the apparent densities increased to 2690 kg/m^3^ and 2680 kg/m^3^, respectively. Further analysis of Figure 7b reveals that for C-RCA, the increase in apparent density after carbonation is 8.69% and 6.09% for 5–10 mm and 10–20 mm aggregates, respectively, while for D-RCA, the increase is 5.26% and 5.30% for 5–10 mm and 10–20 mm aggregates, respectively. The findings presented above show that the carbide slag slurry pre-impregnation–carbonation treatment outperforms the direct carbonation treatment in terms of the apparent density of recycled aggregates. The primary explanation is that during the carbonation process, calcium hydroxide is transformed into calcite, which has a higher density and hence contributes to the total rise in aggregate density [31,32]. However, due to the decrease in carbonation efficiency with depth, the calcium carbonate content in the RCA’s top layer increases significantly, but the interior layer remains unchanged [33], explaining the modest increase in apparent density. This conclusion is consistent with the findings of V.G. Papadakis [34], who found that carbonated recycled aggregate improved water absorption and apparent density while also increasing the microhardness of the transition zone of the old interface, resulting in a greater improvement in the physical properties of recycled aggregates. V. Morales-Florez [35] found that using CO_2_ carbonation to modify recycled aggregate improved concrete performance, providing theoretical support for the use of pre-immersed carbonation treatment technology in engineering applications.

#### 3.1.4. Preliminary Cost Analysis

There are significant differences in the cost composition between natural aggregates and C-RCA. The procurement cost of natural aggregates mainly includes expenses for mining, processing, and transportation, while the main costs of C-RCA focus on crushing, screening, transportation, and CO_2_ gas procurement. Since the raw materials of recycled aggregates are sourced from recycled construction waste, raw material costs are excluded; the calcium carbide slag, an industrial solid waste produced during acetylene production, was kindly provided free of charge by Haohua Company for this experiment. The costs for crushing and screening are determined based on local standard surveys and consultations with recycled aggregate producers. The costs for CSS pre-immersed and carbonation treatment are estimated according to CO_2_ procurement prices and relevant labor expenses. The specific cost breakdown is shown in Table 5.

### 3.2. Effect of Pre-Immersed Carbonated CSS Recycled Aggregate on Mechanical Properties of Recycled Concrete

#### 3.2.1. Workability

**Figure 8:**
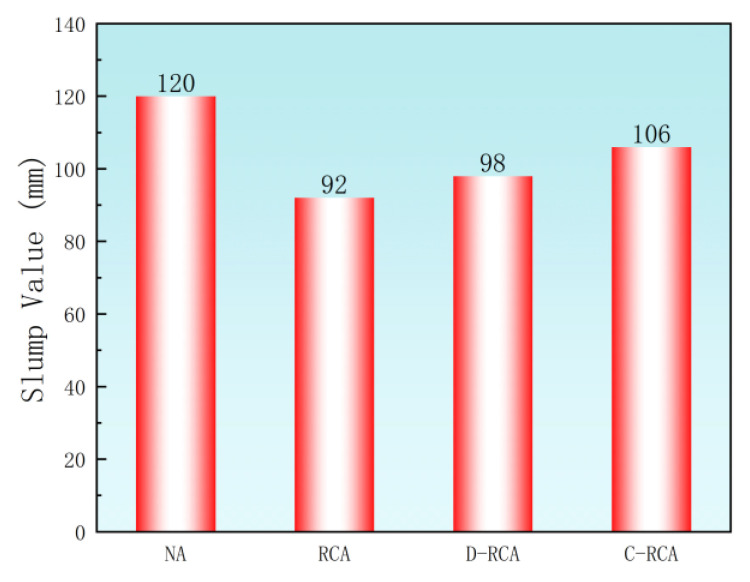
Slump value.

Figure 8 demonstrates the slump test results of NAC, RAC, D-RAC, and C-RAC. The tests show that the slump of both D-RAC and C-RAC is significantly higher compared to RAC. This is attributed to the fact that D-RCA and C-RCA have a lower water absorption rate than RCA, which absorbs less water during the mixing process, thus improving concrete flow. Significantly, despite these variations, all measured slump values fall within the acceptable range specified by China’s GB 50164-2011 [36] standard, confirming adequate workability and validating the practical applicability of these mixtures in construction projects. This finding validates the effectiveness of the mixture design, which successfully reconciles constructability with mechanical performance. It is important to note that, due to the study’s emphasis on mechanical properties, water-reducing agents were not utilized; however, for applications demanding higher slump values, incorporating such admixtures provides an efficient approach to adjusting workability.

#### 3.2.2. Compressive Properties

**Figure 9:**
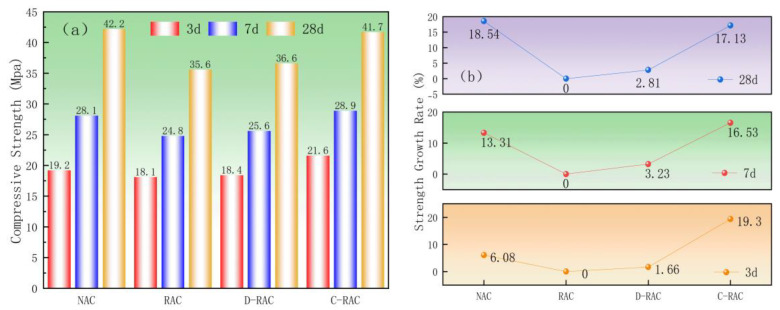
Effect of pre-immersed carbonation of CSS on compressive strength of recycled concrete: (**a**) compressive strength, (**b**) strength growth rate.

Figure 9 depicts the effect of pre-immersed CSS carbonation-treated recycled coarse aggregate on the compressive strength of 100 mm in side cubic recycled concrete as the curing age increases. Figure 9a shows that, when compared to recycled concrete (RAC), the compressive strength of directly carbonated recycled aggregate concrete (D-RAC) increased by 0.3, 0.8, and 1.0 MPa at curing ages of 3, 7, and 28d, respectively, and the compressive strength of CSS pre-immersed carbonated recycled aggregate concrete (C-RAC) increased by 3.5, 4.1, and 6.1 MPa. According to Figure 9b, after a curing age of 28 days, the compressive strength of C-RAC increased by 17.13% when compared to RAC, which is about equal to the cubic compressive strength of normal concrete (NAC), but the compressive strength of D-RAC increased by just 2.81%. The experimental results show that recycled aggregate treated with carbide slag slurry pre-immersed with carbon improves the compressive properties of recycled concrete. Carbonated pre-immersed slag slurry recycled aggregate concrete has enough Ca(OH)_2_ and hydrated calcium silicate (C-S-H) to react with CO_2_ to form CaCO_3_ and silica gel. This improves the densification and strength of the aggregate due to the better stabilization and filling effect of the reaction products, enhancing the compressive strength of cubes. This reinforces the importance of supplying an additional calcium source prior to the carbonation of recycled material.

The strength development of both NAC and RAC aligns with established trends [37]. Specifically, RAC exhibited a 15.6% reduction in 28-day compressive strength due to residual mortar porosity, which corresponds with findings reported by Peng [37] and Tabsh [38].

#### 3.2.3. Splitting Tensile Properties

**Figure 10:**
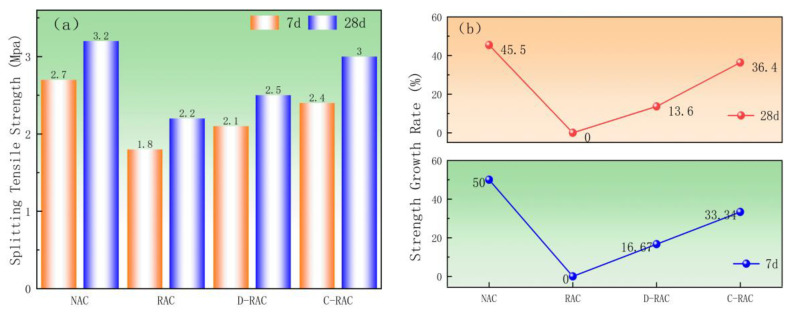
Effect of CSS pre-immersed carbonation treatment on split tensile strength of recycled concrete: (**a**) splitting tensile strength, (**b**) strength growth rate.

To systematically investigate the effect of pre-immersed CSS carbonation treatment and curing age on the splitting tensile strength of recycled concrete, this study tested the mechanical properties of recycled concrete specimens under various treatment conditions, and the test results are shown in Figure 10. As shown in Figure 10a, compared to RAC, D-RAC increased by 0.3 MPa, and C-RAC increased by 0.6 MPa at 7d; D-RAC grew by 0.3 MPa, and C-RAC increased by 0.8 MPa at 28d, suggesting that the split tensile strength of the specimens following the carbonation of prepreg CSS was significantly enhanced. Figure 10b demonstrates that the split tensile strength of C-RAC grew by 36.4% compared to that of RAC at 28d, which is comparable to that of NAC, whereas D-RAC’s split tensile strength increased by just 13.6%. The carbonation reaction added silica gel and CaCO_3_ to the RCA, improving its quality, strengthening the mortar, and reducing the interfacial transition zone. This improved the splitting tensile properties of the pre-immersed CSS carbonated recycled aggregate concrete, as confirmed by SEM microanalysis.

(1) (fts≈0.3fcu2/3)

fts−splitting tensile strength

fcu−compressive strength

Systematic comparison based on the predictive formula from GB/T 50081-2019 (Formula (1)) reveals the following: The measured splitting tensile strength of the NAC group was 3.2 MPa versus a predicted value of 3.6 MPa, with the deviation falling within a reasonable range, indicating the formula’s applicability to normal concrete. For the RAC group, the measured value of 2.2 MPa deviated significantly from the predicted value of 3.2 MPa, and the D-RAC group also showed a large deviation (measured 2.5 MPa vs. predicted 3.3 MPa), demonstrating the limitations of the standard formula for recycled aggregate concrete. In contrast, the C-RAC group exhibited a measured splitting tensile strength of 3.0 MPa compared to a predicted value of 3.6 MPa, showing a reduction in deviation compared to RAC.

#### 3.2.4. Flexural Properties

**Figure 11:**
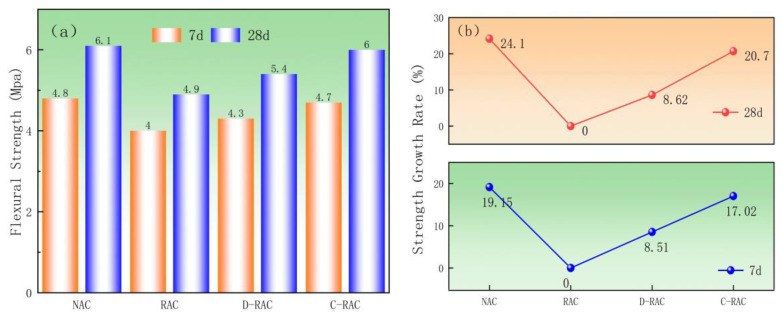
Effect of pre-immersed carbonation treatment of CSS on flexural strength of recycled concrete: (**a**) flexural strength, (**b**) strength growth rate.

Figure 11 shows the change in flexural strength of recycled concrete with the carbonation treatment method and curing age. The fracture resistance of recycled aggregate concrete is lower than that of ordinary concrete. This is because the crushing value of RCA is higher, the internal cracks and pores are larger, and the old mortar attached to the surface is looser, resulting in poor bonding between the old and new cement paste in recycled concrete, which is prone to fracture when subjected to external loading. Figure 11a shows that, when compared to RAC, the flexural strength of D-RAC increased by 0.4 MPa and 0.5 MPa at 7 and 28d, while the flexural strength of C-RAC increased by 0.8 MPa and 1.2 MPa, respectively. According to Figure 11b, the flexural strength of C-RAC grew by 20.7% compared to that of RAC at the age of 28 days, which was similar to that of NAC, whereas the flexural strength of D-RAC increased by only 8.62%. The statistics shown above suggest that carbonated recycled aggregate can improve the flexural strength of recycled aggregate concrete, with the carbonation of pre-immersed carbide slurry having a greater positive effect. Carbonated pre-immersed slag slurry recycled aggregate concrete improves its quality, strength, and flexural properties by incorporating silica gel and CaCO_3_ from the carbonation reaction.

(2) $$\left(ff\approx 0.1\sim 0.15fcu\right)$$

ff−flexural strength

According to GB/T 50081-2019 (Formula (2)), a systematic validation of the prediction formula for concrete flexural strength was conducted. The experimental results show that the measured flexural strength of the NAC group was 6.1 MPa, falling within the predicted range (4.2–6.3 MPa), verifying the applicability of this formula for normal concrete. The measured value for the RAC group was 4.9 MPa, within the predicted range (3.6–5.3 MPa). For the D-RAC and C-RAC groups subjected to different pretreatment processes, the measured flexural strengths were 5.4 MPa and 6.0 MPa, respectively. Both values fluctuated within their predicted intervals (3.7–5.49 MPa and 4.2–6.3 MPa). The close agreement between the measured data from all experimental groups and the theoretical predictions fully demonstrates that these experimental results possess good accuracy and reliability.

### 3.3. Microstructural Analysis

#### 3.3.1. XRD Test

**Figure 12:**
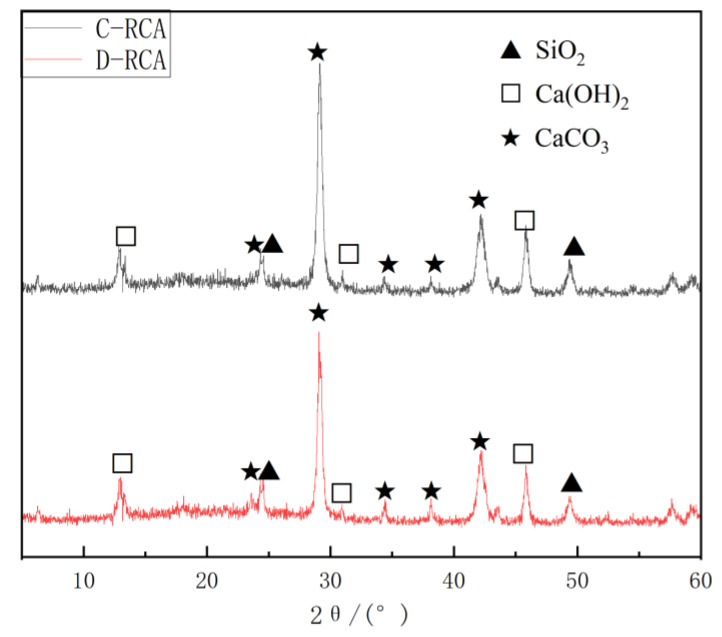
X-ray diffraction pattern of RCA.

Zhang [39] identified a crystalline phase of the cement hydration product Ca(OH)_2_ in RCA. However, the intensity of its diffraction peaks was low, implying that the number of chemicals engaged in the carbonation reaction was minimal. This study gives a theoretical basis for introducing additional calcium sources before carbonation. To investigate the physical phase composition of the carbonation products in this research, the surface of the treated recycled aggregate was studied with X-ray diffraction, and the results are shown in Figure 12. Carbonation enhanced the intensity of CaCO_3_ diffraction peaks in D-RCA and C-RCA, resulting in a matched 2θ of 29.1. C-RCA exhibited more intense CaCO_3_ diffraction peaks than D-RCA. The carbonation of pre-immersed CSS improves the carbonation degree of RCA, resulting in more abundant CaCO_3_ crystals that fill the microcracks and pore structures of aggregates, increasing the density of the internal structure. This synergistic mechanism of carbonation product deposition-filling supports the prior experimental results of aggregate physical characteristics reported in Section 3.1.

#### 3.3.2. SEM Observations

**Figure 13:**
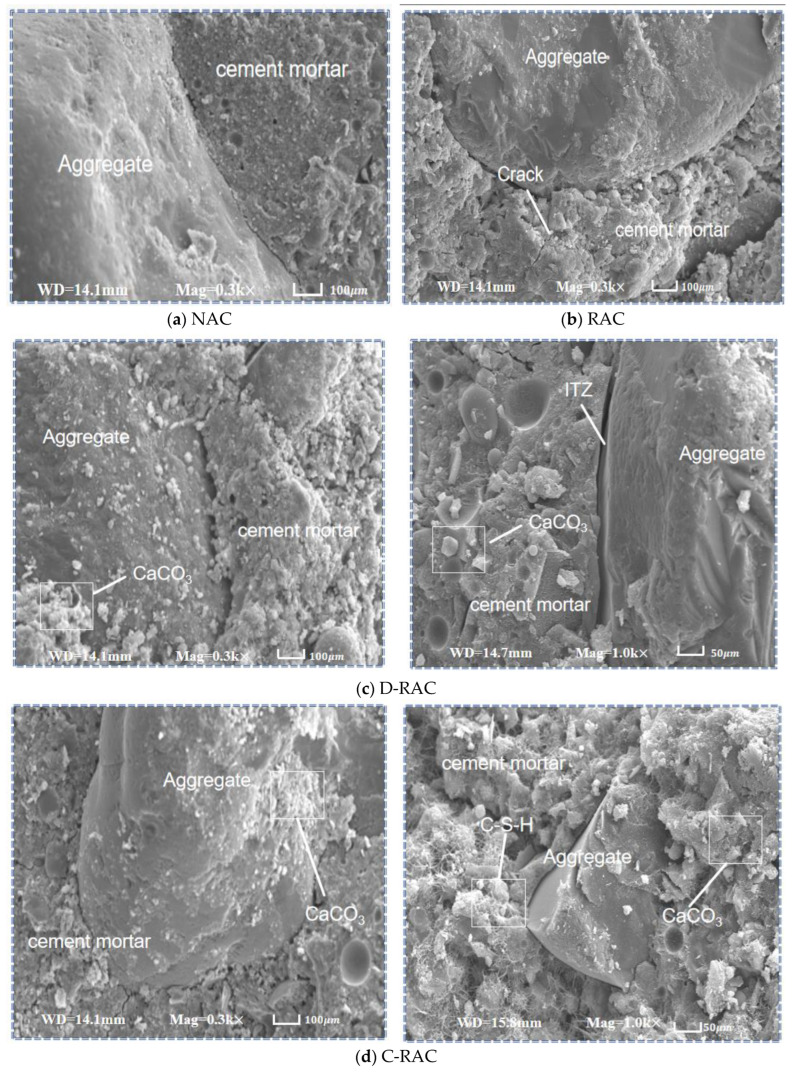
Microstructure of recycled aggregate concrete.

Scanning electron microscopy was used to characterize the microstructure of the recycled concrete, and Figure 13 depicts the microstructure of the ITZ and hydration products between the RCA and the bonded mortar before and during carbonation.

Figure 13a depicts the microstructure of natural aggregate concrete at 300 times magnification, demonstrating that the internal structure of normal concrete is denser and the width of the interfacial transition zone between aggregate and mortar is narrower, resulting in excellent mechanical properties of normal concrete. Figure 13b shows that the internal structure of recycled aggregate concrete is poorly compacted, with more pores and cracks on the surface of the cement paste, between the old and new paste, and between the paste and aggregate, as well as more obvious cracks in the interfacial transition zone, resulting in recycled aggregate concrete’s poor mechanical properties. Figure 13c shows that the strengthening impact of direct carbonation on recycled aggregate is poor; while some carbonation products are produced, there are still more gaps and cracks between the paste and the aggregate. The lack of calcium sources in carbonation leads to lower CaCO_3_ production and unfilled pores. In contrast, Figure 13d shows that the carbonized CSS slurry prepreg recycled aggregate concrete has a denser internal structure, the pores on the slurry’s surface are filled, and there is a good combination between the old and new slurry, the slurry, and the aggregate, and the interfacial transition zone is denser.

The pre-immersed CSS slurry contains a high calcium source for carbonation, creating a favorable environment for the reaction of CO_2_ with Ca(OH)_2_ and hydrated calcium silicate (C-S-H) within the CSS slurry. The carbonation reaction produces CaCO_3_ and silica gel, which improves the densification and strength of the aggregate due to better stabilization and filling effects of the reaction products. This conclusion is supported by the experimental results of concrete’s mechanical properties in the previous Section 2.2.

#### 3.3.3. EDS Analysis

**Figure 14:**
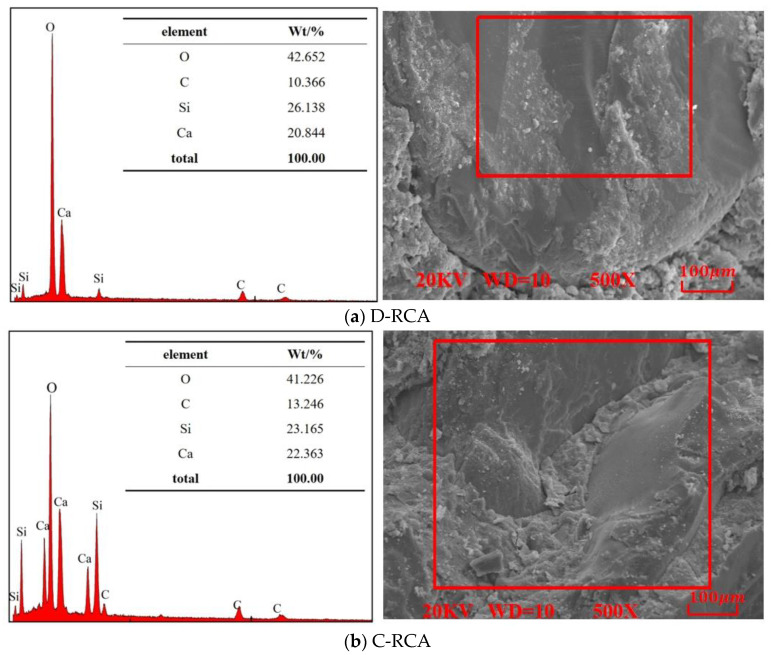
EDS scan of recycled aggregates.

Figure 14 shows the content of each piece retrieved from point scanning. The figure shows that the recycled aggregate comprises elements such as oxygen, silicon, calcium, and carbon. According to B.J. Zhan [40], recycled aggregate carbonation produces CaCO_3_, which fills microcracks and holes at the aggregate mortar interface. The aforementioned EDS analysis results show that the recycled aggregate with pre-immersed carbonation had a higher Ca content than the recycled aggregate with direct carbonation. This suggests that the recycled aggregate with pre-immersed carbonation generates more CaCO_3_ than the recycled aggregate with direct carbonation, demonstrating that it effectively fills the pores and microcracks at the interfacial transition zone and encourages the production of CaCO_3_. This is consistent with the previous mechanical property test results.

## 4. Reinforcement Mechanism of CSS Pre-Immersed Carbonated Recycled Aggregates

The primary causes of recycled concrete aggregate’s (RCA’s) much higher water absorption and crushing value when compared to natural aggregate’s (NA’s) are twofold: first, the aggregate’s structural integrity is greatly diminished by the microcrack network created during crushing; second, the old mortar’s adhesion to the aggregate’s surface creates a loose and porous structure that deteriorates the interfacial transition zone (ITZ) properties.

Research by Zhang J [41] and Xiao J [42] demonstrated that the carbonation reaction is primarily the reaction of CO_2_ with CH and hydrated calcium silicate (C-S-H) to produce CaCO_3_ and silica gel (reaction formulas are shown in Equations (3) and (4)). The solid volume increased by 11.8% following the reaction, which is explained by the fact that the carbonation products filled the pore space, increasing the solid volume. Zhang J [29] demonstrated that this reaction has a dual advantage: on the one hand, the generated calcite (CaCO_3_) and silica gel (SiO_2_.nH_2_O) have excellent stability; on the other hand, the reaction products significantly enhance the aggregate compactness through the pore-filling effect, and the coupling improves the physical properties of the carbonation-treated RCA sufficiently.

(3) $${CO}_{2}+{Ca(OH)}_{2}\to {CaCO}_{3}+H_{2}O$$

(4) $${\left(CaO\right)}_{x}\left({SiO}_{2}\right)\left(H_{2}O\right)+{xCO}_{2}\to {xCaCO}_{3}+{SiO}_{2}n\left(H_{2}O\right)+\left(z-t\right)H_{2}O$$

Yang J [43] discovered that the interfacial transition zone of RAC is relatively weak, which is the primary source of stress damage. Carbonation converts CH and C-S-H to thermally stable CaCO_3_ and silica gel, improving RAC strength through filling. According to Li L [44], C2S and C3S are the main mineral components in silicate cement. C3S has high hydration activity and produces C-S-H and CH. As the reaction progresses, C-S-H undergoes a decalcification reaction with CO_2_ to generate amorphous silica gel to fill in the pores, thus improving the early mechanical strength of concrete.

In this experiment, the recycled aggregate was pre-immersed with CSS slurry to supplement it with extra calcium hydroxide (CH), providing an appropriate calcium supply for the subsequent carbonation process. Pan G [45] used the thermogravimetric method to examine the content of carbonatable compounds (primarily CH) in the aggregate before carbonation, and the results showed that the aggregate itself had a very low content of carbonatable compounds, confirming the need for an additional calcium source. In this experiment, the CH in the CSS slurry was able to infiltrate the pores and microcracks of the recycled aggregates, providing continuous reactants for the carbonation reaction. As the carbonation reaction progresses, a large amount of calcium carbonate precipitation is produced, and these products effectively fill the aggregate’s pore structure, significantly reducing water absorption while increasing apparent density, thereby optimizing the aggregate’s physical properties.

**Figure 15:**
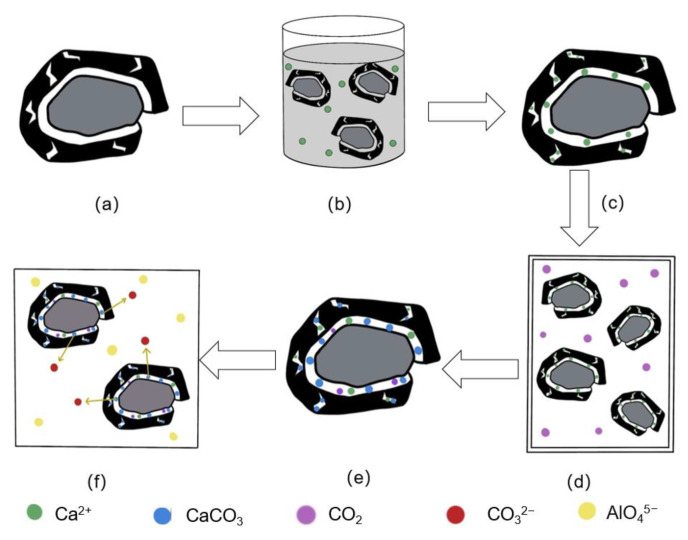
Mechanism of CSS pre-immersed carbonation to improve performance of recycled aggregates. (**a**) RCA. (**b**) CSS pre-immersed recycled aggregate. (**c**) Recycled aggregates after pre-immersed CSS treatment. (**d**) Recycled aggregate after carbonation pretreatment. (**e**) C-RCA. (**f**) C-RAC.

From the experimental results, it can be seen that the performance enhancement impact of the pre-immersed carbonated recycled aggregate is most clear, and its enhancement process is depicted in Figure 15. The recycled aggregate generated a large number of microcracks and pores during the pre-crushing process (a); after the pre-immersing of CSS slurry (b), a large amount of externally added calcium source was provided for the aggregate (c), and these Ca^2+^ were free on the surface and inside of the aggregate, which improved the degree of the subsequent carbonation reaction (d) and the subsequent carbonation reaction(e). Carbonation produced a significant amount of CaCO_3_ and silica gel, which filled the aggregate’s pores, microcracks, and ITZs. CaCO_3_ in recycled concrete from modified aggregate slowly dissolved and released CO_3_^2−^ (f). At the same time, aluminate ions in the cement matrix migrated to the vicinity of calcite, where they reacted to form calcium monocarboaluminate hydrate (Mc), providing more nucleation sites for the growth of C-S-H on the surface of RCA [46], resulting in localized densification around the aggregate and improving the mechanical properties of RAC.

## 5. Conclusions

This study uses CSS pre-immersed and carbonation technology to improve the performance of recycled aggregates and then produces recycled concrete with treated recycled aggregates. We systematically investigated the optimization mechanism of the physical properties of recycled aggregate by CSS pre-impregnation–carbonation treatment, the improvement effect on the mechanical properties of recycled concrete, and the change rule of the microstructure before and after the treatment. The main findings of the study are as follows:

(1) The CSS pre-immersing carbonation treatment significantly improves the physical properties of recycled coarse aggregates. This is because pretreatment with carbide slag slurry substantially enhances carbonation reaction efficiency and improves its surface modification effects. Compared to the untreated aggregate, the physical properties of the recycled aggregate after the synergistic treatment of CSS pre-immersed carbonation are more prominent; 5–10 mm and 10–20 mm recycled aggregate water absorption decreased by 30.07% and 26.70%, respectively; the crushing value decreased by 18.6% and 17.2%; and the apparent density increased by 8.69% and 6.09%.
(2) Pre-immersion of CSS carbonated recycled aggregate can significantly improve the mechanical characteristics of recycled concrete. The reason is that carbonation enhances the strength of old/adhered mortar, improves bonding at the matrix interface, and promotes the formation of denser concrete structures. Pretreatment with CSS provides additional calcium ions to recycled aggregates, establishing optimal conditions for subsequent carbonation. Consequently, the synergistic CSS pretreatment-carbonation treatment delivers the most significant performance improvement in recycled aggregate concrete. CSS pre-immersed carbonated recycled aggregate concrete improved its 3d, 7d, and 28d compressive strength by 3.5, 4.1, and 5.1 MPa, splitting tensile strength by 0.6 and 0.8 MPa in 7d and 28d and flexural strength by 0.8 and 1.2 MPa in 7d and 28d, respectively.
(3) Microstructural analysis indicated that the carbonation alteration had a dual enhancing mechanism for concrete densification. XRD test results show that carbonation of pre-immersed CSS slag slurry significantly increased the height of the characteristic CaCO_3_ peak in recycled aggregate compared to untreated recycled concrete. The reaction products were filled in the ITZ, resulting in an effective enhancement of the ITZ strength. SEM analysis revealed that CO_2_ reacted with CH and C-S-H to produce calcite-type calcium carbonate and amorphous silica gel in concrete made from recycled aggregates pre-immersed with CSS slurry. This effectively filled the microcracks and pores in the aggregate mortar interface’s transition zone (ITZ). EDS studies revealed that the prepreg recycled aggregate increased CaCO_3_ production and efficiently filled microcracks and pores in the interfacial transition zone.

## Figures and Tables

**Table 1:** Chemical composition of the cement (wt%).

CaO	SiO_2_	Al_2_O_3_	Fe_2_O_3_	MgO	K_2_O	Na_2_O
49.49	31.5	9.7	3.93	3.31	1.31	0.76

**Table 2:** Physical properties of aggregates.

Physical Property	5–10 mm NCA	10–20 mm NCA	5–10 mm RCA	10–20 mm RCA
Apparent density (kg/m^3^)	2821	2740	2465	2545
Water absorption rate (%)	0.88	0.62	8.68	4.79
Crushing value (%)	19.98	17.8	25.97	23.2
Los Angeles Abrasion Loss Rate (%)	18	15	31	28
Moisture content (%)	1.8	1.8	1.95	1.95
Packing density (kg/m^3^)	1405	1425	1230	1260
Flakiness Index (%)	10.6	7.2	4.6	3.5
Elongation Index (%)	12.4	10.8	7.1	6.0

**Table 3:** Carbide slag composition (wt%).

Chemical Composition	CaO	SiO_2_	Al_2_O_3_	Fe_2_O_3_	MgO	K_2_O	Na_2_O	Impurity	Total
Ratio	71.12	2.41	0.54	0.4	0.31	0.26	0.15	24.81	100

**Table 4:** Mix proportions of concrete (kg/m^3^).

Specimens	Cement	FA	NCA	RAC	D-RAC	C-RAC	Water
NAC	350	650	1000	0	0	0	175
RAC	350	650	700	300	0	0	175
D-RAC	350	650	700	0	300	0	175
C-RAC	350	650	700	0	0	300	175

**Table 5:** Detailed cost (CNY/ton).

Group	Raw Material Cost	Crushing and Screening Treatment Cost	CSS Pre-Immersed and Carbonation Treatment	Total
NA	80	0	0	80
C-RCA	0	8–12	60	68–72

## Data Availability

The original contributions presented in this study are included in the article. Further inquiries can be directed to the corresponding author.

## Abbreviations

The following abbreviations are used in this manuscript:

CSS	carbide slag slurry
FA	fine aggregate
NCA	Natural Concrete Aggregate
RCA	recycled concrete aggregate
D-RCA	Direct Carbonation Recycled Concrete Aggregate
C-RCA	Carbide Slag Slurry Pre-impregnation Carbonated Recycled Concrete Aggregate
NAC	natural aggregate concrete
RAC	recycled aggregate concrete
D-RAC	Direct Carbonation Recycled Aggregate Concrete
C-RAC	Carbide Slag Slurry Pre-impregnation Carbonated Recycled Aggregate Concrete
